# Cell-Mediated Immune Responses to *in vivo*-Expressed and Stage-Specific *Mycobacterium tuberculosis* Antigens in Latent and Active Tuberculosis Across Different Age Groups

**DOI:** 10.3389/fimmu.2020.00103

**Published:** 2020-02-11

**Authors:** Mariateresa Coppola, Raquel Villar-Hernández, Krista E. van Meijgaarden, Irene Latorre, Beatriz Muriel Moreno, Esther Garcia-Garcia, Kees L. M. C. Franken, Cristina Prat, Zoran Stojanovic, Maria Luiza De Souza Galvão, Joan-Pau Millet, Josefina Sabriá, Adrián Sánchez-Montalva, Antoni Noguera-Julian, Annemieke Geluk, Jose Domínguez, Tom H. M. Ottenhoff

**Affiliations:** ^1^Department of Infectious Diseases, Leiden University Medical Center, Leiden, Netherlands; ^2^Institut d'Investigació Germans Trias i Pujol, CIBER Enfermedades Respiratorias, Universitat Autònoma de Barcelona, Barcelona, Spain; ^3^Servei de Neumología Hospital Universitari Germans Trias i Pujol, Institut d'Investigació Germans Trias i Pujol, CIBER Enfermedades Respiratorias, Universitat Autònoma de Barcelona, Barcelona, Spain; ^4^Unitat de Tuberculosi de Drassanes, Hospital Universitari Vall d'Hebron, Barcelona, Spain; ^5^Serveis Clínics, Unitat Clínica de Tractament Directament Observat de la Tuberculosi, CIBER de Epidemiología y Salud Pública (CIBEREESP), Madrid, Spain; ^6^Servei de Pneumologia, Hospital Sant Joan Despí Moises Broggi, Sant Joan Despí, Spain; ^7^Infectious Diseases Department, Vall d'Hebron University Hospital, PROSICS Barcelona, Universitat Autònoma de Barcelona, Barcelona, Spain; ^8^Grupo de Estudio de Micobacterias (GEIM), Sociedad Española de Enfermedades Infecciosas y Microbiología Clínica (SEIMC), Madrid, Spain; ^9^Malalties Infeccioses i Resposta Inflamatòria Sistèmica en Pediatria, Unitat d'Infeccions, Servei de Pediatria, Institut de Recerca Pediàtrica Hospital Sant Joan de Déu, CIBER de Epidemiología y Salud Pública (CIBERESP), Madrid, Spain

**Keywords:** IVE-TB antigens, *Mycobacterium tuberculosis (Mtb)*, TB, LTBI, cytokines, cell responses

## Abstract

A quarter of the global human population is estimated to be latently infected by *Mycobacterium tuberculosis* (*Mtb*), the causative agent of tuberculosis (TB). TB remains the global leading cause of death by a single pathogen and ranks among the top-10 causes of overall global mortality. Current immunodiagnostic tests cannot discriminate between latent, active and past TB, nor predict progression of latent infection to active disease. The only registered TB vaccine, Bacillus Calmette-Guérin (BCG), does not adequately prevent pulmonary TB in adolescents and adults, thus permitting continued TB-transmission. Several *Mtb* proteins, mostly discovered through IFN-γ centered approaches, have been proposed as targets for new TB-diagnostic tests or -vaccines. Recently, however, we identified novel *Mtb* antigens capable of eliciting multiple cytokines, including antigens that did not induce IFN-γ but several other cytokines. These antigens had been selected based on high *Mtb* gene-expression in the lung *in vivo*, and have been termed *in vivo* expressed (IVE-TB) antigens. Here, we extend and validate our previous findings in an independent Southern European cohort, consisting of adults and adolescents with either LTBI or TB. Our results confirm that responses to IVE-TB antigens, and also DosR-regulon and Rpf stage-specific *Mtb* antigens are marked by multiple cytokines, including strong responses, such as for TNF-α, in the absence of detectable IFN-γ production. Except for TNF-α, the magnitude of those responses were significantly higher in LTBI subjects. Additional unbiased analyses of high dimensional flow-cytometry data revealed that TNF-α+ cells responding to *Mtb* antigens comprised 17 highly heterogeneous cell types. Among these 17 TNF-α+ cells clusters identified, those with CD8+TEMRA or CD8+CD4+ phenotypes, defined by the expression of multiple intracellular markers, were the most prominent in adult LTBI, while CD14+ TNF-α+ myeloid-like clusters were mostly abundant in adolescent LTBI. Our findings, although limited to a small cohort, stress the importance of assessing broader immune responses than IFN-γ alone in *Mtb* antigen discovery as well as the importance of screening individuals of different age groups. In addition, our results provide proof of concept showing how unbiased multidimensional multiparametric cell subset analysis can identify unanticipated blood cell subsets that could play a role in the immune response against *Mtb*.

## Introduction

Tuberculosis (TB) kills more than 4,000 persons per day and is the most life-threatening disease caused by a single infectious agent, *Mycobacterium tuberculosis* (*Mtb*) (1). Although one fourth of the global population is estimated to be latently infected (2), none of the current immunodiagnostics can discriminate between recent and past *Mtb* infection, or predict risk of possible TB progression in LTBI (3–5). Although neonatal vaccination with Bacillus Calmette-Guérin (BCG), the only licensed TB vaccine, protects infants against severe forms of TB, it is unable to prevent active pulmonary TB in adults and adolescents, such that it has little impact on *Mtb* transmission (6). To boost or replace BCG, multiple TB vaccine candidates have been proposed and are being evaluated in different higher end preclinical or clinical phase 1 and phase 2 studies (7). Recently one subunit vaccine candidate, M72/AS01E, induced significant protection from developing TB in a LTBI population (8, 9). Although the numbers of prevented cases were relatively low as yet (10 TB cases in the M72/AS01E group vs. 22 TB cases in the placebo group, a vaccine efficacy of 54%), the outcome of this phase IIb trial supports the power of rational *Mtb* antigen-based subunit TB vaccine development.

Ideally, *Mtb* proteins selected as targets for new TB vaccines should be expressed during active *Mtb* lung infection and efficiently trigger immune effector cells capable of controlling or clearing the infection without inflicting major tissue damage. Recently, we identified a new class of *Mtb* antigens, named IVE-TB, encoded by *Mtb* genes that were highly and consistently expressed in the lung of susceptible (C3HeB/FeJ) as well as resistant (C57BL/6J) mice following aerosol *Mtb* (Erdman) challenge (10, 11). Besides their high expression in *Mtb* infected lungs, these IVE-TB proteins constitute an attractive group of novel candidate antigens for multiple other reasons (11): (i) they are conserved among 219 *Mtb* clinical isolates and thus cover a wide array of *Mtb* strains; (ii) they have high homology with BCG and thus have potential as booster vaccines; (iii) they have high homology with pathogenic mycobacteria, including *M. leprae* and NTM and thus have potential as broader vaccines; (iv) they contain a large number of epitopes predicted to bind to HLA-Ia and HLA-II alleles (coverage of 85% of the human population); (v) they are well-recognized by immune blood cells from *Mtb* exposed subjects (as shown in 37 *Mtb* exposed individuals); and (vi) they elicit immune cells that are producing multiple cytokines besides IFN-γ, a key cytokine known to be necessary but not sufficient in conferring protection against TB (12).

IFN-γ has been used as the main readout to study classical *Mtb* antigens such as latency antigens (DosR regulon encoded antigens, HBHA), resuscitation-promoting factors (Rpfs), and secreted antigens (such as ESAT-6 and CFP-10) (5, 13). Latency antigens are thought to be mostly expressed during latent stages of *Mtb* infection (14), while Rpf proteins appear to be functionally required in the transition from a dormant to an actively replicating state of *Mtb* (15). In support of this, several *Mtb* stage specific antigens were recognized more strongly by IFN-γ producing cells from LTBI than from TB patients and therefore have been proposed as novel *Mtb* antigen specific tools to differentiate latent *Mtb* infection from active TB disease (16–20). Additionally, although to only a limited extent, the recognition of some of these *Mtb* stage specific antigens has been assessed by immune parameters other than IFN-γ, including IL-12, IP-10, IL-10, TNF-α (20–25).

To date, the most extensively characterized cellular subsets participating in the response to *Mtb* antigens have been mono- or poly-functional CD4+ T cells producing IFN-γ, TNF-α, and/or IL-2 (26). However, it remains understudied which other cell subsets recognizing *Mtb* antigens may be involved in the overall response. Recent evidence for example has highlighted a role for NK cells (27) as well as ILC3 (28) in protective immunity to TB. In this study, we first validated the recognition of several recently identified *Mtb* antigens by multicomponent cytokine signatures in an independent cohort of LTBI and TB patients. The magnitude of those responses was higher in latently *Mtb* infected subjects. Additionally, the use of high dimensional single cell data analysis revealed numerous clusters of antigen specific TNF-α+ cells, uncovering immunological heterogeneity in cellular subsets responding to *Mtb* antigens in different age groups of LTBI and TB.

## Materials and Methods

### Study Setting and Patients Recruitment

In this study, whole blood samples were collected from 20 adults (age range = 27–51) and 15 adolescents (age range = 13–17) with pulmonary active TB (*n* = 18) or latent *Mtb* infection (LTBI) (*n* = 17) (Table 1). Active TB patients were determined by a compatible X-ray, positive *Mtb* sputum culture and/or positive PCR. In adolescents the TB diagnosis was also supported by a positive tuberculin skin test (TST) and a known TB contact. LTBI cases were defined by a positive TST and/or QuantiFERON-TB Gold In tube (QFN-G-IT) test. Donors were recruited from six centers located in Barcelona, Spain: Germans Trias i Pujol University Hospital, Unitat de Tuberculosi Vall d'Hebron-Drassanes, Serveis Clínics TB Directly Observed Treatment Unit, Vall d'Hebron University Hospital, Sant Joan Despí Moises Broggi Hospital and Sant Joan de Déu Barcelona Children's Hospital. Among all the subjects included in this study only one (TB21) had a record of drug intake unrelated to TB treatment (the antidepressant Citalopram). The study was approved by the Ethics Committee of all participating centers (reference CEIC: PI-15-073) (http://www.ceicgermanstrias.cat/) and performed following the guidelines and regulations. For each participant a written informed consent was collected together with a detailed questionnaire about clinical and demographic data of the study participant.

**Table 1:** Demographic and clinical characteristics of all patients included in the study.

	**Overall** **(*****n*** **=** **35)**		**Adults** **(*****n*** **=** **20)**		**Adolescents** **(*****n*** **=** **15)**	
	**TB** **(*n* = 18)**	**LTBI** **(*n* = 17)**	**TB** **(*n* = 12)**	**LTBI** **(*n* = 8)**	**TB** **(*n* = 6)**	**LTBI** **(*n* = 9)**
Age, average (years) ± *SD*	32.7 ± 14.1	26.8 ± 13.5	41.1 ± 8.6	40 ± 6.1	15.8 ± 1.2	15.0 ± 1.0
Gender (%)						
Female	5 (27.8)	10 (58.8)	2 (16.7)	4 (50.0)	3 (50.0)	6 (66.7)
Male	13 (72.2)	7 (41.2)	10 (83.3)	4 (50.0)	3 (50.0)	3 (33.3)
Country of birth (%)						
High TB burden^a^	2 (11.1)	0 (0.0)	0 (0.0)	0 (0.0)	2 (33.3)	0 (0.0)
Low TB burden	16 (88.9)	17 (100.0)	12 (100.0)	8 (100.0)	4 (66.7)	9 (100.0)
BCG vaccination (%)	11 (61.1)	5 (29.4)	7 (58.3)	5 (62.5)	4 (66.7)	0 (0.0)
Known TB contact (%)	4 (22.2)	14 (82.3)	2 (16.7)	5 (62.5)	2 (33.3)	9 (100.0)
TST >15 mm (%)						
≥15 mm (%)	5 (27.8)	10 (58.8)	1 (8.3)	5 (62.5)	4 (66.7)	5 (55.6)
<15mm (%)	5 (27.8)	5 (29.4)	3 (25.0)	1 (12.5)	2 (33.3)	4 (44.4)
Unknown/not tested	8 (44.4)	2 (11.8)	8 (66.7)	2 (25.0)	0 (0.0)	0 (0.0)
QFN-G-IT						
Positive	17 (94.4)	16 (94.1)	11 (91.7)	7 (87.5)	6 (100.0)	9 (100.0)
Negative	1 (5.6)	0 (0.0)	1 (8.3)	0 (0.0)	0 (0.0)	0 (0.0)
Unknown/not tested	0 (0.0)	1 (5.9)	0 (0.0)	1 (12.5)	0 (0.0)	0 (0.0)
Prophylaxis (%)						
<30 days	–	14 (82.3)	–	6 (75.0)	–	0 (0.0)
>30 days	–	1 (5.9)	–	1 (12.5)	–	8 (88.9)
Average (days) ± *SD*	–	22.9 ± 7.1	–	27.4 ± 8.2	–	19.0 ± 2.5
No prophylaxis	–	2 (11.8)	–	1 (12.5)	–	1 (11.1)
Anti-TB treatment (%)						
<30 days	6 (33.3)	–	4 (33.3)	–	2 (33.3)	–
>30 days	10 (55.6)	–	6 (50.0)	–	4 (66.7)	–
Average (days) ± *SD*	40.2 ± 17.0	–	37.6 ± 18.0	–	48.8 ± 33.2	–
No treatment	2 (11.1)	–	2 (16.7)	–	0 (0.0)	–

a *About 150 incident cases per 100,000 population*.

*BCG, bacillus Calmette-Guérin; LTBI, latent tuberculosis infection; QFN-G-IT, QuantiFERON Gold In-Tube; SD, standard deviation; TB, tuberculosis; TST, tuberculin skin test*.

### Recombinant Proteins

A total of 59 *Mtb* recombinant proteins, previously identified by different *Mtb* antigen discovery approaches (13), were tested in this study (Table 2). As described previously (11), *Mtb* genes were amplified by PCR from genomic H37Rv DNA and cloned by Gateway technology (Invitrogen, Carlsbad, CA, USA) in a bacterial expression vector containing, overexpressed in *Escherichia coli* (*E. coli*) BL21 (DE3) and purified. Gel electrophoresis and western blotting with an anti-His Ab (Invitrogen) and an anti-*E. coli* polyclonal antibody (a gift of Statens Serum Institute, Copenhagen, Denmark) were used to check the size and purity of the recombinant proteins. Rv0287-Rv0288, Rv2346c-Rv2347, and Rv3614-Rv3615 were produced as fusion proteins to mirror the pairwise dependent secretion pathway followed by T7S systems. All recombinant proteins were tested to exclude protein-non-specific T cell stimulation and cellular toxicity (11).

**Table 2:** List of *Mtb* antigens included in this study.

**List**	**Rv number**	**Function**	**Category**	**Ref**
1	Rv0066	oxalosuccinate decarboxylase	IVE-TB	(11)
2	Rv0287/ Rv0288	EsxG/EsxH	IVE-TB	(11)
3	Rv0383c	Possible conserved secreted protein	IVE-TB	(11)
4	Rv0423c	ThiC	IVE-TB	(11)
5	Rv0440	GroEL2	IVE-TB	(11)
6	Rv0467	icl1	IVE-TB	(11)
7	Rv0468	FadB2	IVE-TB	(11)
8	Rv0470c	PcaA	IVE-TB	(11)
9	Rv0501	GalE2	IVE-TB	(11)
10	Rv0640	RplK	IVE-TB	(11)
11	Rv0642c	MmaA4	IVE-TB	(11)
12	Rv0645	MmaA1	IVE-TB	(11)
13	Rv0824c	Acyl-desaturase DesA1	IVE-TB	(11)
14	Rv0826	Conserved hypothetical protein	IVE-TB	(11)
15	Rv0991	Conserved serine rich protein	IVE-TB	(11)
16	Rv1038c	EsxJ	IVE-TB	(11)
17	Rv1131	PrpC	IVE-TB	(11)
18	Rv1221	SigE	IVE-TB	(11)
19	Rv1284	Beta-carbonic anhydrase	IVE-TB	(11)
20	Rv1390	RpoZ	IVE-TB	(11)
21	Rv1479	MoxR1	IVE-TB	(11)
22	Rv1738	Conserved hypothetical protein	IVE-TB/latency antigen	(11, 14)
23	Rv1791	PE19	IVE-TB	(11)
24	Rv1792	EsxM	IVE-TB	(11)
25	Rv1846	BlaI	IVE-TB	(11)
26	Rv1872	IldD2	IVE-TB	(11)
27	Rv1980c	Mpt64	IVE-TB	(11)
28	Rv2007	FdxA	IVE-TB	(11)
29	Rv2031	HspX	IVE-TB	(11)
30	Rv2215	DlaT	IVE-TB	(11)
31	Rv2245	KasA	IVE-TB	(11)
32	Rv2346c/ Rv2347c	EsxO/EsxP	IVE-TB	(11)
33	Rv2382	Polyketide synthetase mbtC	IVE-TB	(11)
34	Rv2431c	PE25	IVE-TB	(11)
35	Rv2461	ClpP1	IVE-TB	(11)
36	Rv2626	Hrp1	IVE-TB/latency antigen	(11, 14)
37	Rv2657c	PhiRv2 prophage protein	IVE-TB	(11)
38	Rv2710	SigB	IVE-TB	(11)
39	Rv2873	Mpt83	IVE-TB	(11)
40	Rv2941	28	IVE-TB	(11)
41	Rv3048c	R1F protein	IVE-TB	(11)
42	Rv3052	FadB4	IVE-TB	(11)
43	Rv3407	VapB47	IVE-TB	(11)
44	Rv3462	IF-1 infA	IVE-TB	(11)
45	Rv3583c	Possible transcription factor	IVE-TB	(11)
46	Rv3614/ 3615	/EspC	IVE-TB	(11)
47	Rv3615	EspC	IVE-TB	(11)
48	Rv3616^*^	EspA	IVE-TB	(11)
49	Rv3846	SodA	IVE-TB	(11)
50	Rv3865^*^	EspF	IVE-TB	(11)
51	Rv3874/ Rv3875	CFP10/ESAT6	IVE-TB/secreted antigens	(5, 11)
52	Rv0867c	RpfA	Rpf	(15)
53	Rv1009	RpfB	Rpf	(15)
54	Rv1733c	Rv1733c	latency antigen	(14)
55	Rv2032	acg	latency antigen	(14)
56	Rv2034	Rv2034	IVE-TB	(11)
57	Rv2389c	RpfD	Rpf	(15)
58	Rv2450c	RpfE	Rpf	(15)
59	Rv3353c	Rv3353c	IVE-TB	(11)

*Rpf, resuscitation-promoting factors; Ref, reference(s)*.

* *Antigens included in a patented TB vaccine candidate*.

### Whole Blood Assay

Within 3 h of collection, heparinized venous blood was diluted 1:10 in AIM-V medium (Invitrogen, Breda, the Netherlands). Samples were incubated (450 μl/well) in 48-well-plates at 37°C, 5% CO_2_, with 50 μl antigen solution (final concentration of 10 μg/ml). After 6 days, 200 μl of the supernatants was collected from each well and frozen in aliquots at −20°C until further analysis.

### Multiple Cytokine Array and Analysis of Diluted Whole Blood Supernatant

As it has been recommended by a cross-laboratory evaluation of multiplex bead assays, in this study we used one multiplex kit from the same manufacturer (29). The R&D TM premixed Multi-analyte kit (Cat #: 1415903) was used to measure the concentrations of eight analytes (IL-13, IL-22, IL-17A, IFN-γ, induced protein 10 [IP-10 (CXCL10)], IL-10, GM-CSF, and TNF-α) in diluted whole blood culture supernatants according to manufacturer's instructions. These cytokines were selected to validate and extend our previously published findings (11). To rule out batch effects, for each multiplex run samples with different disease status and age were always mixed. Data were acquired using Luminex 200 (Luminex Corp., Austin, TX) and analyzed using Bio-Plex Manager software 6.0 (Bio-Rad Laboratories), as previously described (11). The median background values of unstimulated samples were: 14 pg/ml (GM-CSF, LTBI = 14 pg/ml and TB = 14 pg/ml), 47 pg/ml (IFN-γ, LTBI = 47 pg/ml and TB = 47 pg/ml), 3 pg/ml (IL-10, LTBI = 3 pg/ml and TB = 4 pg/ml), 5 pg/ml (IL-17A, LTBI = 4 pg/ml and TB = 5 pg/ml), 10 pg/ml (IL-22, LTBI = 12 pg/ml and TB = 9 pg/ml), 17 pg/ml (IP-10, LTBI = 10 pg/ml and TB = 20 pg/ml), and 3 pg/ml (TNF-α, LTBI = 3 pg/ml and TB = 4 pg/ml). Values outside the upper (ULOQ) or lower (LLOQ) limits of quantification were set as the values of the analyte detection limits. Due to the high LLOQ for IL-13 (median = 177 pg/ml), the values detected for this cytokine did not fall in the linear part of the standard curve and therefore were not further analyzed.

### IL-32 ELISA

Based on new reports describing the possible role of IL-32 in protection against TB (30, 31), we decided to assess the IL-32 alpha concentration in supernatants, using the DuoSet ELISA (R&D Systems, Catalog #: DY3040-05) according to the manufacturer's instructions. Samples were diluted 2-fold in reagent diluent (R&D Systems, Catalog #: DY995). The optical density (O.D.) was acquired by Varioskan Flash (Thermo Electron Corporation) using the SkanIt software version 2.4.1. Data were linearized by plotting the log of the human IL-32 concentration (pg/ml) vs. the log of the O.D. and the fit line was determined by regression analysis using GraphPad Prism (version 7.0). The concentration read from the standard curve was multiplied by the dilution factor of 2. The median background value of unstimulated samples was 2 pg/ml for both TB ad LTBI groups.

### Blood Processing for Whole Blood Intracellular Staining (WB-ICs) Assay

As previously described (32, 33), venous blood was collected from 15 study participants (described in **Figure 3A**) in sodium heparin tubes and, within 60 min (34), 1 ml of blood was transferred into Sarstedt tubes containing either AIM-V medium alone or combined with a pool of *Mtb* antigens (Rv1131, Rv2461, and Rv3616c, selected based on the multiplex results), or PPD, in the presence of co-stimulants (anti-CD28 and anti-CD49d, each at 1 μg/ml, BD Biosciences, Erembodegem, Belgium). The IVE-TB proteins were tested at a final concentration of 10 μg/ml while PPD (Statens Serum Institut, Copenhagen, Denmark) was used at a final concentration of 5 μg/ml.

After 3 h of incubation in a water bath set at 37°C, Brefeldin A (3 μg/ml; Sigma-Aldrich, Zwijndrecht, the Netherlands) and Monensin (1:1,000; BD Biosciences) were added. Samples were then transferred back to the water bath, programmed to switch off after 12 h. Samples remained in the water bath and EDTA was added (2 mM final concentration) 9 h later and incubated for 15 min at room temperature in order to detach adherent cells. Erythrocytes were lysed and white blood cells fixed with FACS lysing solution (BD Biosciences). The fixed cells were pelleted and cryopreserved in 1 ml of FCS with 10% dimethyl sulfoxide (DMSO).

### Flow Cytometry Reagents

Fixed whole-blood samples were thawed in a water bath at 37°C for 2 min and stained with a 14-color FACS panel in permeabilization solution (Fix&Perm cell permeabilization kit, An Der Grub BioResearch GMBH, Susteren, the Netherlands). The 14-color FACS panel included: CD3-PE-TexasRed (clone S4.1) (Thermo Fischer), CD4-Pacific Blue (clone RPA-T4), CD28 PerCP-Cy5.5 (clone L293), CD8-PECy5 (clone RPA-T8), CD14-HorizonV500 (clone RPA-T8), IFN-γ-AlexaFluor700 (clone B27) (all BD Biosciences), CD45RA-Brilliant Violet 650 (clone HI100), CD27-Brilliant Violet 605 (clone O323), TNF-α-APC-Cy7 (clone MAb11), IL-10-PE-Cy7 (clone JES3-9D7), IL-13-PE (clone JES10-5A2) (all BioLegend), IL-17-A-FITC (clone eBio17B7), IL-22-PerCP-eFluor710 (clone 22 URTI) (all eBioscience), IL-32-allo-phycocyanin (APC) (clone 373821) (R&D). Samples were acquired on a BD LSRFortessa using FACSDiva software (version 6.2, BD Biosciences) with compensated parameters.

### Data Analysis

Significant differences (*p* < 0.05) between cytokine levels in stimulated and unstimulated samples were evaluated by Mann–Whitney *U*-test corrected for multiple comparison (FDR, Benjamini-Hochberg test correction). The R package “phenotypicForest” was used to construct the polar histogram (35). After dividing by the background-values, i.e., AIM-V medium, for each response in each donor, differences between cytokine levels in LTBI donors and TB patients were assessed by Mann-Whitney *U*-test (*p* < 0.05) and median log_2_ fold-changes [log2FC(LTBI/TB)> 1 or < −1]. Results are reported only for *Mtb* antigens that induced increased cytokine levels compared to the background-values. Multiple R-squared derived by a linear model was used to compare responses between different age groups (https://www.rdocumentation.org/packages/stats/versions/3.6.1/topics/lm).

Flow cytometry data were analyzed using FlowJo v10. Results from single-stained and unstained mouse/rat κ beads were used to calculate compensations. Cell doublets were excluded using forward scatter-area vs. forward scatter-height parameters. After debris exclusion, TNF-α+ cells were gated and FCS files of stimulated samples (antigen pool and PPD) were exported. FACS data were transformed using hyperbolic arcsin with a cofactor of 150 directly within Cytosplore^+HSNE^ (36). Then, for each donor and condition the gated events were randomly down-sampled to the same number of TNF-α+ events as the sample with the lowest number of TNF-α+ cells (*n* = 153), to ensure equal representation of all samples. Next, a HSNE analysis was performed on a total of 3,366 TNF-α+ cells after defining the markers used for the similarity computation (CD45, CD3, CD4, CD28, CD8, CD14, IFN-γ, CD27, IL-10, IL-13, IL-17A, IL-22, and IL-32). We used the standard parameters for the hierarchy construction; number of random walks for landmark selection: *N* = 100, random walk length: *L* = 15, number of random walks for influence computation: *N* = 15, number of scale = 2. For any clustering that occurred the GMS grid size was set to *S* = 256 (36). The iterations chosen of the HSNE analysis were 1,000. We clustered the data with a kernel size sigma of 30 on the overview level without manual modifications. Cell clusters were inspected using the integrated heatmap visualization. The median abundance (i.e., median cell frequency) of the cells composing each cluster was calculated per each group of donors and conditions (heatmap generated in Morpheus: https://software.broadinstitute.org/morpheus). Based on the median fluorescence intensity reported in the Supplementary Table 3, manual gating using OMIQ (www.omiq.ai) was performed to investigate seven populations, each of those representing a best approximation of the main clusters discussed in the paper (Supplementary Figure 1A). GraphPad Prism 8.2.1 was used to depict the frequencies of these cell populations for each donor (Supplementary Figure 1B).

## Results

### IVE-TB *Mtb* Antigens, *Mtb* DosR-regulon, and Rpf Antigens Activate Multiple-Cytokine-Producing Blood Cells of *Mtb* Exposed Subjects

**Figure 1:**
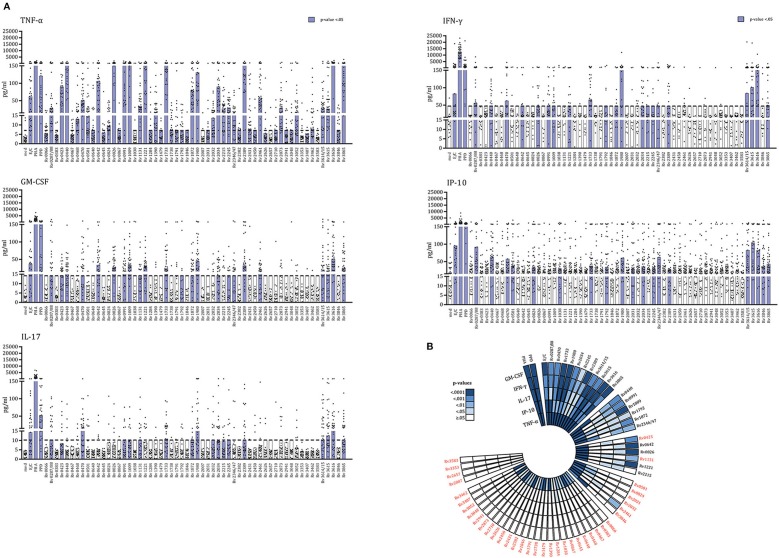
IVE-TB *Mtb* antigens, *Mtb* DosR-regulon, and Rpf antigens activate multiple-cytokine-producing blood cells of *Mtb* exposed subjects. The levels of eight cytokines were measured in diluted whole blood supernatants from a cohort of *Mtb* exposed individuals (*n* = 35) after 6 days stimulation with either IVE-TB, DosR, Rpf (10 μg/ml), control antigens ESAT6/CFP10 (E/C) (10 μg/ml), PPD (5 μg/ml), or the positive control, mitogen PHA (2 μg/ml). The statistical significance of the differences between cytokine levels in stimulated and unstimulated samples was evaluated by Mann-Whitney *U*-test with FDR multiple test correction for IL-13, IL-22, IL-17A, IFN-γ, IP-10, IL-10, GM-CSF, IL-32, and TNF-α. **(A)** Results are shown only for TNF-α, GM-CSF, IP-10, IFN-γ, and IL-17A for which significant differences were found among single *Mtb* antigens and unstimulated samples. Each dot represents a donor. Bars indicate medians. Purple bars indicate significantly increased responses compared to the unstimulated samples with a *p* < 0.05 (Mann-Whitney *U*-test with FDR multiple test correction). **(B)** The polar histogram displays the different *p*-values found among single *Mtb* antigens (or control) and unstimulated samples. Stimuli are ordered clockwise as follows: controls and *Mtb* antigens grouped according to how many cytokines were induced. These groups of antigens are separated by interruptions of the histogram. In red are indicated *Mtb* antigens that induced cytokines other than IFN-γ.

We recently described a new class of *Mtb* antigens, termed IVE-TB antigens, that were recognized by blood cells of *Mtb* exposed subjects from two small Northern European LTBI cohorts (*n* = 37). IVE-TB antigens not only elicited canonical IFN-γ but also alternative cytokines including GM-CSF, IP-10, IL-13, TNF-α, and IL-17 (11). To formally and independently validate these findings, we here tested these antigens in a Southern European cohort of 35 *Mtb* exposed individuals, which included LTBI as well as TB patients (Table 1). In addition to IVE-TB proteins we also included two latency antigens (Rv1733c and Rv2032) and four resuscitation-promoting factors (Rv0867c, Rv1009, Rv2389c, and Rv2450c), accumulating to a total of 59 *Mtb* proteins (Table 2). These highly immunogenic DosR- and Rpfs- antigens, known to distinguish latent *Mtb* infection from active TB disease based on IFN-γ production levels as previously reported (13, 16–20), were here included to investigate whether they could also induce additional cytokines. To allow a comparison with our previously published study (11), we selected the same cytokines (IL-13, IL-22, IL-17A, IFN-γ, induced protein 10 [IP-10 (CXCL10)], IL-10, GM-CSF, and TNF-α) and assay (multiplex on the supernatants of diluted whole blood incubated for 6 days with single or fusion *Mtb* antigens) as read-out, with only the new addition of IL-32 (rationale explained in the methods section). When measuring these multicomponent cytokine signatures in the supernatants of diluted whole blood (incubated for 6 days with single or fusion *Mtb* antigens), secretion of TNF-α, GM-CSF, IFN-γ, IP-10, and IL-17A (but not of IL-13, IL-22, IL-10, and IL-32) was significantly increased in stimulated compared to unstimulated samples (Mann–Whitney *U*-test with FDR multiple test correction) (Figure 1A, Supplementary Table 1). In agreement with our previous findings, not all antigens induced the same type of cytokines response (Figure 1A). However, most of the antigens studied (*n* = 54) induced strong TNF-α production. Interestingly, out of 59 *Mtb* proteins, 30 were significantly recognized by cells producing two to five cytokines, which not always included canonical IFN-γ (Figure 1B).

Together these results extend, and most importantly, validate our previous findings in a completely independent cohort of donors, by demonstrating multi- rather than single- component cytokine signatures in the response to IVE-TB antigens, and for the first time also to DosR and Rpf stage specific *Mtb* antigens. Importantly, we confirm immune recognition of *Mtb* antigens in the absence of detectable IFN-γ production in a significant number of cases (Figure 1B).

### LTBI Individuals' and TB Patients' Blood Cells Secrete Different Amounts of Cytokines in Response to IVE-TB and Stage Specific *Mtb* Antigens

**Figure 2:**
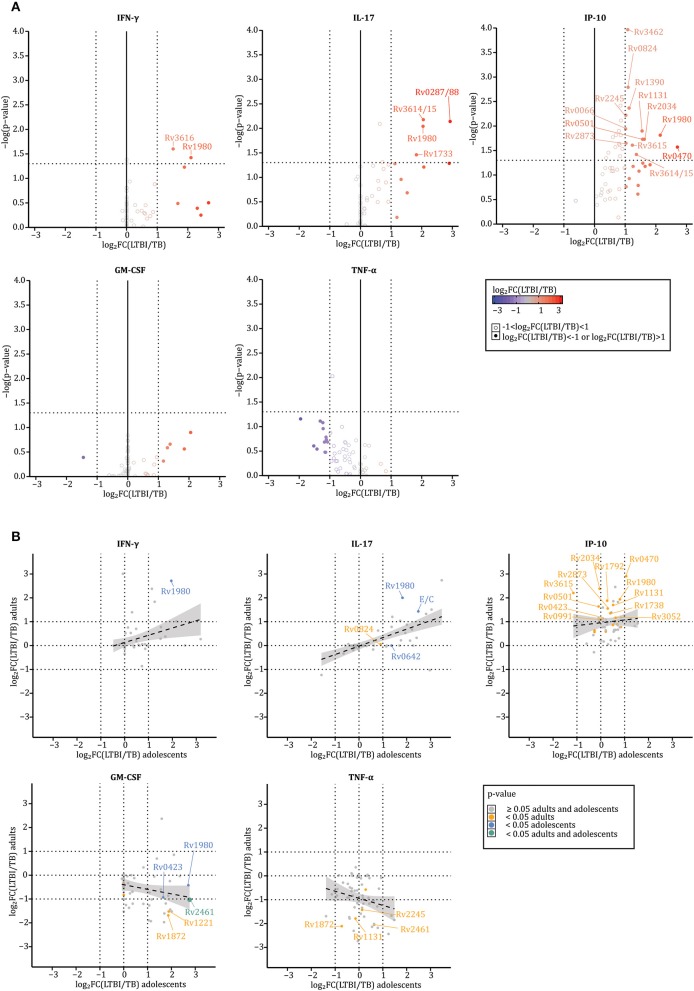
LTBI individuals' and TB patients' blood cells secrete different amounts of cytokines in response to IVE-TB and stage specific *Mtb* antigens. The levels of nine cytokines were measured in diluted whole blood supernatants after 6 days stimulation with either IVE-TB, DosR, Rpf antigens (10 μg/ml), ESAT6/CFP10 (E/C) (10 μg/ml), PPD (5 μg/ml), or PHA (2 μg/ml). The cytokine responses were compared between LTBI donors (*n* = 17) and TB patients (*n* = 18). This comparison is displayed for five cytokines, TNF-α, GM-CSF, IFN-γ, IP-10, and IL-17A, which were found to be significantly increased in stimulated samples compared to unstimulated ones (Figure 1). Within each donor, prior to further analysis, the pg/ml values detected in response to every antigen were normalized to the background-values, i.e., divided by the medium, and then log_2_ transformed. Results are reported only for *Mtb* antigens that induced increased cytokine levels compared to the background-values. **(A)** Cytokine differences were assessed by Mann–Whitney *U*-test (significant for *p*-values < 0.05) and by median log_2_ fold-changes [log_2_FC(LTBI/TB) > 1 or <-1). *P*-values and median log_2_ fold-changes are reported on the y and x axes, respectively. Thresholds for *p*-values and median log_2_ fold-changes are indicated by dotted lines intersecting the axes (x:−1 and 1; y: 1.30103). Closed circles define when the log_2_FC(LTBI/TB) is >1 (red) or <−1 (blue), such that closed red circles define antigens inducing higher responses in LTBI donors than TB patients, while blue circles depict antigens inducing higher responses in TB patients than LTBI subjects. Open circles define when the log_2_FC(LTBI/TB) is >−1 and <1 (gray). **(B)** Differences between LTBI and TB patients were analyzed separately among adults (TB *n* = 8; LTBI *n* = 12) and adolescents (TB *n* = 9; LTBI *n* = 6). X and y-axes indicate the median log_2_ fold-changes in adolescents and adults, respectively. The color-coded dots indicate *p*-values: ≥0.05 in adults and adolescents (gray), <0.05 in adults (orange), <0.05 in adolescents (blue), and <0.05 in adults and adolescents (green). The gray area around the regression line (dotted line) represents the range in which the true regression line lies at a 95% level of confidence. Both in **(A,B)**, the Rv numbers are coupled to antigens recognized differently by LTBI and TB according to both *p*-values and median log_2_ fold-changes.

Next, we assessed whether the *in vitro* cytokine profiles in response to the selected 59 *Mtb* antigens (Table 2) were similar for stimulated blood cells from LTBI subjects (*n* = 17) or active (*n* = 18) TB patients (Table 1). This comparison was performed for five cytokines, TNF-α, GM-CSF, IFN-γ, IP-10, and IL-17A, that had been found to be significantly increased in *Mtb* antigen stimulated samples above (Figure 1). Prior to analysis, the concentrations detected in response to every antigen within each donor were normalized to background-values, i.e., divided by the medium concentration, and log_2_ transformed. Cytokine differences were assessed by Mann–Whitney *U*-test (significant for *p* < 0.05) and by median log_2_ fold-changes [log_2_FC(LTBI/TB) > 1 or < −1]. Results described below focus only on those *Mtb* antigens that induced significantly different responses as confirmed by both *p*-values and median log_2_ fold-changes (Figure 2, Supplementary Table 2). Of note, although the TB group consisted of untreated patients and patients treated for less or more than 30 days, we did not find significant differences in the level of cytokine secreted among those groups possibly in part due to the small samples size (*p* > 0.05 for all conditions according to a Kruskal–Wallis *H*-test, Supplementary Table 2).

Of all 59 *Mtb* antigens, 16 were preferentially recognized by blood cells from LTBI donors (Rv0066, Rv0287/0288, Rv0470, Rv0501, Rv0824, Rv1131, Rv1390, Rv1733, Rv1980, Rv2034, Rv2245, Rv2873, Rv3462, Rv3615, Rv3616, and Rv3614/3615) (Figure 2A). Except for TNF-α, the cytokines detected in response to *Mtb* antigen stimulation were consistently higher in blood samples from LTBI individuals compared to TB patients (Figure 2A). Of note, the recognition of these 16 antigens by samples from LTBI was primarily mediated by IP-10 production (Figure 2A). Interestingly, this changed upon age-subgroup analyses comparing adults (TB *n* = 8; LTBI *n* = 12) and adolescents (TB *n* = 9; LTBI *n* = 6) (Figure 2B). Although IFN-γ and IL-17A were abundantly secreted by blood cells of LTBI donors independent of age (multiple R-squared derived by linear models: 0.1037 and 0.4192, for IFN-γ and IL-17, respectively), differences in IP-10 responses between LTBI and TB were less marked in adolescents (multiple R-squared derived by linear models: 0.009575) (Figure 2B, upper plots). While the highest TNF-α responses were found in adult TB patients (multiple R-squared derived by linear models: 0.04714), GM-CSF responses were higher in adolescent LTBI donors than in adolescent TB patients; they were also higher in adult TB patients than adult LTBI subjects (multiple R-squared derived by linear models: 0.05153) (Figure 2B, lower plots). Interestingly, these contrasting responses were consistent following stimulation with specific antigens such as Rv2461 or Rv1872 (Figure 2B), lending further validity to these observations.

Collectively, the data show that blood cells from LTBI subjects and TB patients produce qualitatively and quantitatively different cytokines in response to stimulation with *Mtb* antigens. The data, although based on a limited number of donors, also suggest that certain cytokine responses such as IP-10, GM-CSF, and TNF- α can differ between age groups.

### Quantitative and Qualitative Differences in Specific TNF-α+ Responding Cellular Subsets Between LTBI and TB Patients

**Figure 3:**
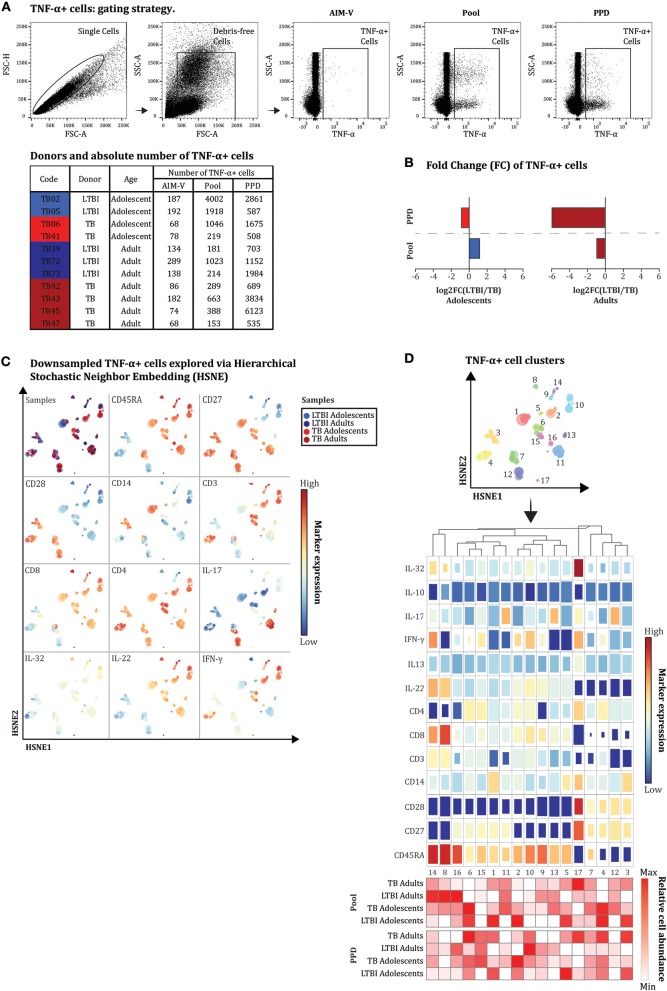
Quantitative and qualitative differences are present in specific TNF-α+ cell subsets between LTBI and TB patients. Intracellular cytokine staining was performed on whole blood (ICS-WB) of a limited number of *Mtb* exposed individuals recruited in this study (*n* = 11). Samples were left unstimulated or stimulated for 12 h with a pool of three *Mtb* antigens (Rv1131, Rv2461, and Rv3616c) or PPD, in the presence of (T cell) co-stimulants (anti-CD28 and anti-CD49d). After staining cryopreserved fixed blood cells with an extensive 14-color FACS panel, TNF-α+ cells were quantified in each sample. **(A)** The gating strategy is shown for one sample (TB02) to illustrate the selection of single debris-free TNF-α+ cells in unstimulated (AIM-V i.e., medium) and stimulated (antigen pool or PPD) sample. In the left upper table, the number of TNF-α+ cells is indicated for each donor. **(B)** The difference in the absolute number of TNF-α+ cells between LTBI and TB was defined by median log_2_ fold change [log_2_FC(LTBI/TB)] and calculated separately in adolescents and adults. Blue or red bars represent log_2_FC(LTBI/TB) >0.5 or <-0.5, respectively. **(C)** For each of the 11 donors, TNF-α+ cells responding to the antigen pool and PPD were used as input in Cytosplore. Input cells were randomly downsampled to 153 events leading to a total of 3,366 TNF-α+ cells to explore via hierarchical stochastic neighbor embedding (HSNE). In the upper left HSNE plot, blue, and red dots indicate cells isolated from LTBI or TB patients (color tonality distinguishes adolscents from adults). In the other 11 HSNE plots the colored dots indicate the expression range of 11 singular cellular markers on the cells analyzed (high expression, red; medium expression, yellow; low expression, blue). **(D)** By applying a Gaussian mean-shift clustering to the FACS data, 17 distinct TNF-α+ cell clusters (displayed in the HSNE plot, upper panel) were defined by a unique combination of expressed markers as shown by the heatmap (middle panel). Differences in the size of colored squares indicate the contribution of each marker in defining the specific cell subset, while colors indicate the relative expression of each cellular marker compared to all the other markers (red indicates high; blue indicates low). Lower heatmap (lower panel) shows the proportion of cells composing each cluster per age group of donors (LTBI and TB) and stimuli (PPD and antigen pool). The numbers in the last row refer to the cell clusters.

To further characterize the functional blood cells involved in the recognition of *Mtb* antigens in a manner unbiased with respect to phenotype, we focused on TNF-α+ cells, since TNF-α was the most abundant cytokine found in the supernatant of diluted whole blood in response to *Mtb* antigen stimulation (Figure 1). To address this question in the same samples as used in the cytokine analyses, we performed intracellular cytokine staining on whole blood (ICS-WB) as previously described by others (32, 34, 37). Due to restriction in blood collection, the ICS-WB could be performed only for a limited number of donors (*n* = 11). Samples were left unstimulated or stimulated over night with a pool of three *Mtb* antigens (Rv1131, Rv2461, and Rv3616c), that had been selected prior to commencing the study on the basis of previous results (11), or PPD. Interestingly, two antigens of the pool (Rv1131 and Rv2461) were among those recognized differently by LTBI and TB patients of different age groups in the cytokine screening (Figure 2B). After staining cryopreserved fixed blood cells with a 14-color FACS panel, all TNF-α+ cells were enumerated in each sample (Figure 3A). Already after 12 h of stimulation, TB patients had larger numbers of TNF-α+ cells [quantified by log_2_FC(LTBI/TB)] in response to PPD compared to LTBI subjects, after correction for background values (Figure 3B). The same trend was observed in response to the *Mtb* antigen pool but only in the adult group, thus partially mirroring the amount of TNF-α protein secreted in supernatants of cells stimulated 6 days with single antigens (Figure 2B).

We next imported the TNF-α+ cells from all stimulated conditions (both with the antigen pool or with PPD) into Cytosplore, and analyzed all data using hierarchical stochastic neighbor embedding (HSNE) (36). HSNE landmarks revealed a clear heterogeneity in the memory cell compartment, distinguishing central memory phenotype like subsets (CD45RA–CD27+CD28+) from effector (CD45RA+CD27+/–CD28+/–) and terminally differentiated effector (CD45RA+CD27–CD28–) memory like subsets (Figure 3C). By applying a Gaussian mean-shift clustering to the flow cytometry data, 17 distinct cell clusters were defined by unique combinations of expressed markers, ranging from 22 to 454 cells per cluster (Figure 3D, upper panel). Hierarchical clustering of the heat map revealed two major groups, corresponding either to the absence or presence of CD45RA (Figure 3D, middle panel). The first major group, composed of cells lacking CD45RA but expressing CD28, clustered in 3 main subpopulations: co-expressing either CD14 but not CD3 [cluster 3, number of cells in the cluster (*N*) = 310], co-expressing CD3 and CD4 (cluster 4, *N* = 352), or expressing IL-17 (cluster 12, *N* = 235) (Figure 3D, middle panel). A fourth CD45RA negative cluster, mostly characterized by increased IL-32 and IFN-γ expression was also identified but since it was defined based on a very low number of cells (cluster 17, *N* = 22) (Figure 3D, middle panel), it is not further described here. The second major group was characterized by a progressively higher expression of CD45RA, combined with differential expression of CD27 molecules, co-expressing either CD4 (cluster 6, *N* = 219; cluster 15, *N* = 125 and cluster 4, *N* = 352), CD8 (cluster 8, *N* = 92; cluster 9, *N* = 93; and cluster 14, *N* = 87) or both (cluster 2, *N* = 196 and cluster 10, *N* = 335) and several intracellular markers including IL-32, IL-17A, IFN-γ, and IL-22 but not IL-10 and IL-13 (Figure 3D). The phenotype of three cell clusters expressing IL-17 could not be fully captured and better defined by the markers included in our analysis (cluster 11, *N* = 454; cluster 12, *N* = 235; and cluster 13, *N* = 88) (Figure 3D, middle panel). Two cell clusters were only defined by different expression of CD45RA, CD27, and CD28 but not in relation to any of the cytokines measured (cluster 16, *N* = 87; cluster 7, *N* = 302) (Figure 3D, middle panel).

The proportion of cells (median relative abundance), derived from LTBI subjects and TB patients, which contributed to the above cell clusters was different between adolescents and adults (Figure 3D, lower panel). More specifically, in our analysis TNF-α+ CD8+ (CD3+CD4–) cells with a poly-functional (IL-22, IFN-γ, and IL-32) terminal effector memory-like phenotype were mainly found in LTBI adults although their presence did not seem to require antigen specific stimulation (clusters 8, 9, and 14 Figure 3D, lower panel; Supplementary Figure 1, Supplementary Table 3). By contrast, TNF-α+ cells co-expressing CD14 (cluster 1, *N* = 301; cluster 3, *N* = 310; and cluster 5, *N* = 68; Figure 3D, lower panel; Supplementary Table 3) mostly originated from antigen stimulated samples of LTBI adolescents and TB adults while they were almost absent in samples from antigen unstimulated and stimulated TB adolescents and LTBI adults (Supplementary Figure 1). Two TNF-α+ CD4 central memory/effector like clusters moderately co-expressing IFN-γ (clusters 4 and 6) were observed only in antigen stimulated samples of adolescent LTBI and TB patients as well as of adult TB patients two (Figure 3D, lower panel; Supplementary Figure 1, Supplementary Table 3). One cluster of TNF-α+ T cells co-expressing CD4 and CD8 and IFN-γ (cluster 10) was more abundantly present in adults LTBI than in TB patients in response to PPD, but this was not found for adolescents (Supplementary Figure 1).

Despite the limited number of donors these results support the presence of higher numbers of TNF-α+ cell subsets in the blood of adult TB patients compared to adult LTBI subjects in response to specific *Mtb* antigens (IVE-TB antigen pool or PPD). In addition to these quantitative differences, our multi-dimensional analysis, looking beyond the key cytokine IFN-γ, defined phenotypically and functionally different TNF-α+ cell subsets in the blood of LTBI and TB patients. Future studies should confirm and assess the immunological relevance of these cell subsets in larger and different cohorts.

Collectively, our findings confirm and validate the recognition of recently described *Mtb* antigens in an independent South European cohort of LTBI and extend this also to active TB patients. We further confirmed that these responses are defined by multiple cytokines besides IFN-γ in both adults and adolescents. Of note is that, except for TNF-α+, the magnitude of those responses was more pronounced during latent *Mtb* infection, irrespective of age. Additionally, through unbiased analysis of high dimensional single cell data, 17 clusters of *Mtb* antigen-specific TNF-α+ cells of both myeloid and lymphoid origin were defined. This suggests the presence of as yet poorly defined but phenotypically and functionally different *Mtb* antigen-responsive TNF-α+ cell subsets in LTBI subjects and TB patients of different age groups.

## Discussion

*Mtb* antigens selected as targets for TB diagnostic tests or TB vaccines need to be able to activate immune cells in *Mtb* exposed individuals (13). This has classically been studied, at least in humans, by measuring *Mtb* antigen induced IFN-γ production in *in vitro* assays. While useful, sensitive and robust, clearly many other molecules are secreted by immune cells, often in the absence of IFN-γ. Our previous work showed that these molecules include GM-CSF, IP-10, IL-13, TNF-α, and IL-17 (11). This concept was recently also confirmed by others (12). In the current study, we validate and confirm these findings in an independent cohort across different age groups and phases of *Mtb* infection (TB vs. LTBI). These molecules all participate in the immune response against *Mtb* antigens, including several IVE-TB and stage specific antigens. Additionally, quantitative differences in cytokine production were found between LTBI and TB patients after *Mtb* antigen stimulation. Interestingly, high dimensional unbiased single cell data analysis defined multiple clusters of TNF-α+ cells and suggested distinct abundancies in these subsets in LTBI and TB patients across different age groups in response to *Mtb* antigen stimulation. These heterogeneous TNF-α+ cell clusters have potential implications for the identification of correlates of infection and of protection, which are urgently needed in TB. Due to the limited number of subjects studied here, no definitive conclusions can be drawn with regard to the immunological relevance of these cell subsets in relation to clinical outcome of infection. This will need validation and further analyses in future studies, including prospective cohorts.

Although IL-12/IFN-γ axis deficiency results in susceptibility to unusual mycobacterial infections (38, 39), the role of IFN-γ in TB resistance and immunopathology, especially in the lung, is not undisputed (4, 40, 41). In spite of this, most *Mtb* proteins have been proposed as candidate antigens almost exclusively on the basis of their recognition by IFN-γ producing cells obtained from latently infected individuals (LTBI) and TB patients (13, 20). Despite the fact that several *Mtb* proteins triggered different type of cytokines and chemokines, including IL-12, IP-10, IL-10, and TNF-α, these broader immune responses have been examined almost only for candidates that had already been selected by prior IFN-γ screening approaches (20–25, 42). Here, in a new cohort of *Mtb* exposed individuals, we corroborated our previous finding on the added value of TNF-α, GM-CSF, IP-10, and IL-17A in the screening of putative *Mtb* antigens using a non-IFN-γ centric approach (Figure 1).

As for IFN-γ, there is no unequivocal evidence defining IL-17A, GM-CSF, and IP-10 as essential to controlling latent *Mtb* infection (43–47) or active TB disease (48–51). TNF-α, generally associated with tissue damage (42, 52), is also considered a key factor in TB granuloma integrity and protective immunity (53). In our cohort, the concentrations of GM-CSF, IL-17A, IFN-γ, and IP-10 were higher in the stimulated blood cell supernatants from LTBI compared to TB patients (Figure 2A). If LTBI control *Mtb* infection better than TB patients (54), those *Mtb* antigens more strongly recognized by blood cells of LTBI correlate with, and potentially could contribute to the host immune response containing *Mtb*. This rational has led to the identification of several TB vaccine candidates whose efficacy has been proven in animal models (55–57), but not yet tested in humans. It therefore remains to be seen whether there is a difference in efficacy between TB vaccine candidates based on antigens preferentially recognized by LTBI vs. TB patients (7, 8, 58–60).

Despite the fact that most of the cytokines in response to *Mtb* antigens were found more abundantly in LTBI, as expected by previous reports (42, 52) we found TNF-α responses to be higher in TB patients (Figure 2A) and as such correlated with pathology. The role of TNF-α is not unequivocal: on the one hand it has a pivotal role in the host defense against tuberculosis (as proven by an increased risk of active disease following anti- TNF-α therapies), on the other hand its excess is also associated with TB pathogenesis both in animal models and humans (61, 62). Interestingly, when analyzing adults and adolescents separately, this difference was more pronounced in the adult group (Figure 2B). This trend in cytokine secretion between the two age groups was also found for GM-CSF (Figure 2B). Adolescents and adults represent the primary target population for subunit TB vaccination (63). However, the immune response of adolescents is not often analyzed as a separate entity but merged with that found in either children (48, 64) or adults (37). The need for carefully considering different age groups is supported by the M72/AS01E efficacy trial results, which showed that the highest vaccine efficacy in preventing active TB among LTBI was in individuals of 25 years of age or younger, after 2.5 years of follow up (8, 9). This observed age-effect has been explained by possibly different timing of primary *Mtb* infection occurrence. Based on this reasoning, younger LTBI due to a more-recent primary *Mtb* infection would be less likely to have the infection under immune control, and therefore could benefit more from boosting immune responses by the administration of M72/AS01E (8, 9). In our cohort, IFN-γ producing cells were not compromised by active TB disease or age (or time of *Mtb* exposure), since blood cells of all groups were similarly effective in secreting IFN-γ in response to antigens present in the QuantiFERON-TB Gold test [ESAT-6/CFP-10/TB-7.7(p4)] (Table 1) and to other specific *Mtb* antigens (Figure 1). However, such impairment could not be excluded for IFN-γ-independent immune mechanisms, such as for GM-CSF and TNF-α responses.

Finding age-related differences among TB and LTBI might suggest that either the abundance, the functionality or the phenotype of GM-CSF and TNF-α producing cells might be heterogeneous in relation to age. To start addressing this issue we focused first on TNF-α+ cells, since the levels of TNF-α were the highest detectable ones in the supernatants of diluted whole blood in response to antigen stimulation. Although the number of donors was limited (*n* = 11), we found that TNF-α+ cells stimulated with three *Mtb* antigens (Rv1131, Rv2461, and Rv3616c) or PPD were more abundant in TB patients than adult LTBIs (Figure 3A). An opposite trend was seen in adolescent LTBI samples compared to TB patients when stimulated with the pool of *Mtb* antigens (Figure 3B). Of note, this pool contained two antigens (Rv1131 and Rv2461) recognized differently by LTBI and TB patients of different age groups in the cytokine screening (Figure 2B). Therefore, one could speculate that part of the differences found in the secreted cytokine profiles reflected the number of TNF-α+ cells recognizing the pool of *Mtb* antigens (Figure 3A).

More interestingly, by analyzing high dimensional single cell data (36), we observed additional differences in the phenotype and functionality of TNF-α+ cells between age groups. TNF-α+ cells co-expressing CD14 (Cluster 1, 5, and 3; Figure 3D) were mainly found in adolescent LTBIs after stimulation with the *Mtb* antigen pool or PPD while almost absent in adult LTBIs. This is interesting since the major source of TNF in human blood seems to be a subset of pro-inflammatory, non-classical monocytes, which have previously been associated with recent *Mtb* exposure (46, 65).

In contrast, among the cells from adult LTBIs, the TNF-α+ cellular subsets were found to be CD8+ T cells with terminal effector memory (TEMRA) like phenotype co-expressing IL-22, IL-32, and IFN-γ although that did not require *Mtb* antigen stimulation (Clusters 8, 9, and 14; Figure 3D, Supplementary Figure 1). TNF-α+ CD8+ TEMRA cells have been already described in adult LTBI and suggested to play a role in antimicrobial activity against TB reactivation (66, 67). In one of these studies, it was elegantly demonstrated that anti-TNF therapy correlated with lower numbers of TNF-α+ CD8+ TEMRA cells and decreased anti-mycobacterial activity that was reverted by the addition of TNF-α+ CD8+ TEMRA cells (66). Thus, it would be interesting to assess whether there is a link between the “protection associated” TNF-α+ CD8+ TEMRA subset previously described and the ones found in our cohort. One subset co-expressing CD4 and CD8 with IFN-γ (cluster 10) was also mainly formed by cells originating from LTBI adults. Recently (68), a new subset of CD4 CD8 double positive T cells able to produce cytokines and cytolytic markers has been identified in the blood, airways and lung granulomas of *Mtb* infected cynomolgus macaques. Additional studies would be required to clarify the role of those cells in the context of active and latent *Mtb* infection. Larger cohorts studied by advanced multiparametric technologies might help to resolve the cell heterogeneity, especially present in LTBI, found in our as well as in previous studies (69). High-dimensional cytometry analyses have been already harnessed to explore cells from differently exposed *Mtb* adolescents uncovering new cell subsets, including those expressing CD16+ and other populations defined as NK cells, CD27–CD8+ αβ T cells, B cells (27), and ILC3 (28).

Despite the relatively limited number of subjects that could be included, our cell subset-unbiased analysis reveals the presence of heterogeneous TNF-α+ cell subsets associated with responses to *Mtb* antigens as well as PPD. These responses could reflect the spectrum of *Mtb* infection and *Mtb* exposure, but their identity and exact function needs further elucidation (70, 71).

In conclusion, our data validate our previous antigen discovery approach, support the value of assessing broader immune responses than IFN-γ alone at an early stage of *Mtb* antigen discovery, and suggest how in depth unbiased profiling of functional cells recognizing *Mtb* antigens can identify a yet ill-defined cell subsets participating in the immune response against *Mtb*.

## Data Availability Statement

All datasets generated for this study are included in the article/Supplementary Material.

## Ethics Statement

This study was approved by the Ethics Committee of all participating centers (reference CEIC: PI-15-073) (http://www.ceicgermanstrias.cat/) and performed following the guidelines and regulations. For each participant a written informed consent was collected together with a detailed questionnaire about clinical and demographic data of the study participant. Written informed consent to participate in this study was provided by the participants' legal guardian/next of kin.

## Author Contributions

MC designed and performed experiments and analysis and wrote the manuscript. RV-H performed experiments and revised the manuscript. KM designed and performed experiments and revised the manuscript. BM and EG-G performed experiments. KF developed recombinant proteins. CP, ZS, MD, J-PM, JS, AS-M, and AN-J were responsible for the recruitment of donors. RV-H, BM, and EG-G organized sample acquisition and database. AG and IL supervised experiments and revised the manuscript. JD and TO supervised, designed experiments, and revised the manuscript. All authors have read and approved the final manuscript.

### Conflict of Interest

The authors declare that the research was conducted in the absence of any commercial or financial relationships that could be construed as a potential conflict of interest.
