# Supplementary figures and images for: Cell-Mediated Immune Responses to in vivo-Expressed and Stage-Specific Mycobacterium tuberculosis Antigens in Latent and Active Tuberculosis Across Different Age Groups
Source: Front Immunol. 2020 Feb 11;11:103. doi: 10.3389/fimmu.2020.00103 (PMC7026259; doi:10.3389/fimmu.2020.00103)

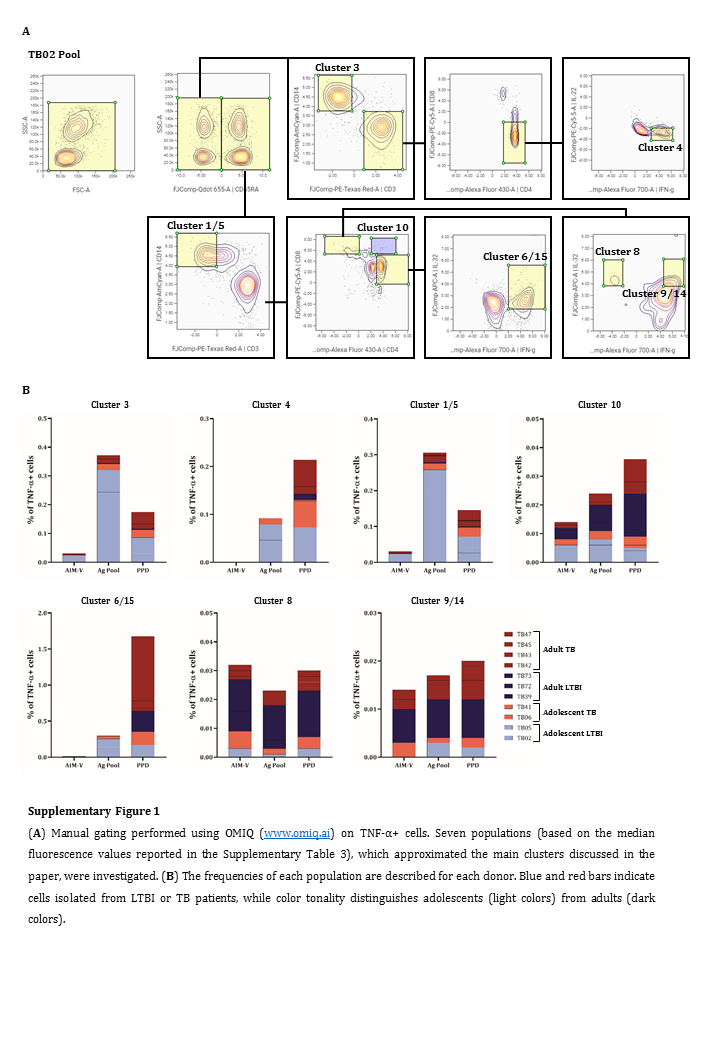

Supplement: Supplementary file 4 [file Image_1.tif]
